# Feasibility and Accuracy of a Dual-Function AR-Guided System for PSI Positioning and Osteotomy Execution in Pelvic Tumour Surgery: A Cadaveric Study

**DOI:** 10.3390/bioengineering12080810

**Published:** 2025-07-28

**Authors:** Tanya Fernández-Fernández, Javier Orozco-Martínez, Carla de Gregorio-Bermejo, Elena Aguilera-Jiménez, Amaia Iribar-Zabala, Lydia Mediavilla-Santos, Javier Pascau, Mónica García-Sevilla, Rubén Pérez-Mañanes, José Antonio Calvo-Haro

**Affiliations:** 1 Musculoskeletal Oncology Division, Department of Orthopaedic Surgery and Traumatology, Hospital General Universitario Gregorio Marañón, Dr. Esquerdo 46, 28007 Madrid, Spaincalvoharo.md.phd@gmail.com (J.A.C.-H.); 2 Lower Limb Division, Department of Orthopaedic Surgery and Traumatology, Hospital General Universitario Gregorio Marañón, Dr. Esquerdo 46, 28007 Madrid, Spain; 3 Advanced Planning and 3D Manufacturing Unit (UPAM3D), Hospital General Universitario Gregorio Marañón, Dr. Esquerdo 46, 28007 Madrid, Spain; 4 Digital Health and Biomedical Technologies, Vicomtech Foundation, Basque Research and Technology Alliance (BRTA), 20009 Donostia-San Sebastian, Spain; 5 Bioengineering Department, Universidad Carlos III de Madrid, 28911 Leganés, Spain

**Keywords:** augmented reality (AR), patient-specific instrument (PSI), head-mounted display (HMD), osteotomy accuracy, surgical navigation, cadaveric study, 3D printing, AR-guided surgery

## Abstract

**Objectives**: Pelvic tumor resections demand high surgical precision to ensure clear margins while preserving function. Although patient-specific instruments (PSIs) improve osteotomy accuracy, positioning errors remain a limitation. This study evaluates the feasibility, accuracy, and usability of a novel dual-function augmented reality (AR) system for intraoperative guidance in PSI positioning and osteotomy execution using a head-mounted display (HMD). The system provides dual-function support by assisting both PSI placement and osteotomy execution. **Methods**: Ten fresh-frozen cadaveric hemipelves underwent AR-assisted internal hemipelvectomy, using customized 3D-printed PSIs and a new in-house AR software integrated into an HMD. Angular and translational deviations between planned and executed osteotomies were measured using postoperative CT analysis. Absolute angular errors were computed from plane normals; translational deviation was assessed as maximum error at the osteotomy corner point in both sagittal (pitch) and coronal (roll) planes. A Wilcoxon signed-rank test and Bland–Altman plots were used to assess intra-workflow cumulative error. **Results**: The mean absolute angular deviation was 5.11 ± 1.43°, with 86.66% of osteotomies within acceptable thresholds. Maximum pitch and roll deviations were 4.53 ± 1.32 mm and 2.79 ± 0.72 mm, respectively, with 93.33% and 100% of osteotomies meeting translational accuracy criteria. Wilcoxon analysis showed significantly lower angular error when comparing final executed planes to intermediate AR-displayed planes (*p* < 0.05), supporting improved PSI positioning accuracy with AR guidance. Surgeons rated the system highly (mean satisfaction ≥ 4.0) for usability and clinical utility. **Conclusions**: This cadaveric study confirms the feasibility and precision of an HMD-based AR system for PSI-guided pelvic osteotomies. The system demonstrated strong accuracy and high surgeon acceptance, highlighting its potential for clinical adoption in complex oncologic procedures.

## 1. Introduction

Pelvic tumor resections are among the most complex orthopedic procedures, requiring precise execution of osteotomies to achieve clear surgical margins while preserving healthy tissue [1]. Conventional approaches often result in suboptimal outcomes, with a success rate of only 52% (95% CI: 37–67) when using free-hand techniques [2]. These challenges are amplified by the pelvis’s irregular anatomy and deep surgical field, making visual and tactile assessment difficult.

To support intraoperative precision, various assistive technologies have been introduced [3]. Computer-assisted navigation systems provide dynamic instrument tracking based on preoperative imaging but require complex setups and an unobstructed line-of-sight to tracking markers—conditions not always guaranteed in a crowded operating room [4,5]. Alternatively, 3D-printed patient-specific instruments (PSIs), designed from patient imaging reconstruction, offer customized cutting paths that improve resection accuracy and have been widely adopted in oncologic orthopedics [6,7,8,9]. Recent clinical analyses have demonstrated that PSIs not only increase the likelihood of achieving tumor-free margins but also contribute to improved relapse-free and overall survival rates [10]. Nevertheless, anatomical variability, soft tissue interference, and the lack of distinct surface landmarks in some bone regions can introduce significant positioning errors, ultimately compromising the planned osteotomy. In response to these challenges, augmented reality (AR) has emerged as a promising solution to guide both PSI placement and osteotomy execution with improved spatial awareness [11].

Among the available AR interfaces, head-mounted displays (HMDs) provide a significant ergonomic advantage by enabling hands-free, surgeon-aligned visualization, surpassing the usability of handheld platforms such as tablets or smartphones [12,13]. 

AR facilitates the superimposition of virtual anatomical models, patient-specific instruments and, potentially, osteotomy planes directly onto the physical surgical field in real time [14]. This capability minimizes reliance on peripheral screens and enhances intraoperative spatial perception. A fundamental component of any AR-assisted system is its registration mechanism. Fiducial markers play a critical role in enabling accurate localization and tracking by serving as known reference points within the physical environment. These markers allow the AR system to reliably align virtual content with real-world anatomy. Costa et al. demonstrated that tracking using ArUco and Vuforia markers could achieve translation and rotation errors as low as 1.36 mm and 0.015°, respectively, underscoring their precision potential for surgical applications [15]. AR has been investigated across multiple orthopedic applications, including pelvic and acetabular procedures, where initial results suggest improved precision and workflow efficiency. Hoch et al. applied AR guidance in cadaveric periacetabular osteotomies, a non-oncologic setting, and reported a mean 3D deviation of 9–17 mm between planned and executed starting points, and a mean angular deviation of 6–7° between planned and performed osteotomies [16]. Additionally, Kimura et al. recently evaluated a pin-less AR navigation system in total hip arthroplasty and found significantly greater accuracy in acetabular cup placement: 90.3% of components were placed within ±5° of the target angle in the AR group compared to 52.6% in the conventional group (*p* < 0.001) [17]. 

Most precision studies with excellent results in this area are limited to proof-of-concept validations in synthetic models, often focusing on isolated steps such as PSI alignment or tool tracking without addressing the full resection workflow. Moreover, few studies have explored AR in complex oncologic resections, and, to our knowledge, none have integrated a dual-function AR system for both PSI placement and osteotomy execution in a cadaveric model. Recent work by García-Sevilla et al. suggested that AR could improve PSI placement accuracy, though their study was confined to two phantom specimens [18]. As part of our research group, Iribar-Zabala et al. conducted a preliminary pre-clinical feasibility study evaluating the same novel AR-assistance software used in the current work. This system uniquely combines guidance for PSI positioning with real-time virtual display of the planned osteotomy plane, enabling dual-function intraoperative assistance. The study demonstrated promising accuracy results and received positive clinician feedback on the proposed workflow [19]. Building upon this internal foundation, we recognized the need for further validation in anatomically realistic environments, which motivated the design of the present cadaveric feasibility study.

This study investigates the feasibility and accuracy of an AR-guided workflow for assisting PSI positioning and osteotomy execution in pelvic tumor surgery. The system, developed for a head-mounted display, provides real-time visualization of both the PSI placement and the osteotomy plane, directly overlaid on the cadaveric anatomy, thus implementing a dual-function intraoperative guidance strategy. Building upon promising results observed in synthetic phantom testing, we hypothesize that, although slightly diminished under realistic anatomical and surgical conditions, the AR-assisted workflow will maintain clinically valuable precision within defined accuracy thresholds.

To evaluate system performance, accuracy thresholds were defined based on clinical relevance and supporting literature. For angular deviation, 0–5° was considered optimal, consistent with high-precision AR and navigation systems in pelvic surgery [17,20]. Deviations of 5–10° were deemed acceptable, as similar errors have not been shown to compromise safety or outcomes, while errors > 10° were classified as unacceptable due to risks to resection margins and reconstruction feasibility [16,21].

We report the maximum error at the osteotomy corner point for translational deviation, representing the worst-case deviation at a critical geometrical anchor. This is a more stringent and clinically meaningful metric in oncologic surgery, where maintaining clear margins is paramount. Thresholds were defined as: excellent (0–3 mm), optimal (4–6 mm), acceptable (7–10 mm), and non-acceptable (>10 mm), aligning with values reported in cadaveric and guided resection studies [9,22].

Although this study focuses on pelvic osteotomies, the proposed AR-guided system is inherently adaptable to other anatomical regions requiring complex and precise bone resections, highlighting its potential as a versatile solution for broader surgical applications.

To the best of our knowledge, this is the first cadaveric study applying augmented reality in the oncological context of pelvic tumor osteotomies using a novel AR-assistance software capable of simultaneously guiding PSI placement and displaying the osteotomy planes.

## 2. Materials and Methods

### 2.1. Design and Fabrication

Ten fresh-frozen left hemipelvis specimens were selected for this study. The left side was chosen for all cases to standardize the approach and avoid bias associated with side dominance. Each specimen underwent a preoperative computed tomography (CT) scan using a 512 × 512 matrix and a pixel size of 0.98 mm to ensure high-resolution anatomical reference for surgical planning and postoperative analysis.

**Figure 1:**
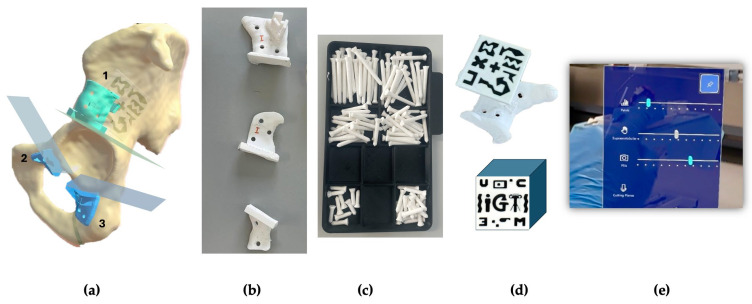
(**a**) Segmented hemipelvis showing the three planned cutting planes. The supraacetabular guide (1) includes a socket for AR-marker placement, while the symphysial (2) and ischial (3) guides are intended for AR-assisted positioning. (**b**) All three guides—supraacetabular, symphysial, and ischial—were 3D-printed using rigid 10k resin. (**c**) Fixation pins (3.5 mm diameter) were also 3D-printed in rigid 10k resin and designed to fit PSI holes prepared with a 3.2 mm drill and 0.6 mm tolerance. (**d**) A 4 × 4 cm AR-marker was printed in high-contrast polylactic acid (PLA) to fit securely into the supraacetabular guide socket. (**e**) The AR application menu displayed on the HoloLens 2 allows the surgeon to toggle the visibility of the biomodel, PSIs, and cutting planes, as well as adjust the transparency of both guides and bone models in real time.

Segmentation of pelvic structures was performed manually using 3D Slicer software (version 16.0, Materialise NV, Leuven, Belgium). A senior orthopedic oncologist defined three osteotomy planes—supraacetabular, ischial, and symphysial—to achieve adequate acetabular resection. Based on these planes, three patient-specific instruments (PSIs) were designed using 3-matic software (Materialise, Belgium) (Figure 1b). Each guide featured fixation holes for 3.5 mm pins (Figure 1c) and was 3D-printed in rigid 10k resin to allow radiological visibility. A socket for an AR-marker was incorporated into the supraacetabular PSI. This location was chosen based on previous published findings identifying it as the most accurate position for manual marker placement [18]. The marker size of 4 × 4 cm was selected based on prior internal testing and previous literature from our group, which demonstrated that this dimension offers an optimal balance between detection robustness, spatial accuracy, and ergonomic integration into the surgical field. The AR-marker measured 4 × 4 cm and was printed with high-contrast polylactic acid (PLA) filament to ensure robust detection using the Vuforia engine (Figure 1d).

The software engineering team developed a novel AR application for the HoloLens 2 headset (Microsoft Corp., Redmond, WA, USA) using Unity and the Mixed-reality Toolkit (MRTK). The application used the Vuforia library for marker detection. It enabled the visualization of 3D holographic models of the PSIs and cutting planes, registered to the physical specimen via the AR marker. Surgeons could toggle visibility and transparency settings via a hand gesture menu during the procedure (Figure 1e).

### 2.2. Experiment Workflow

**Figure 2:**
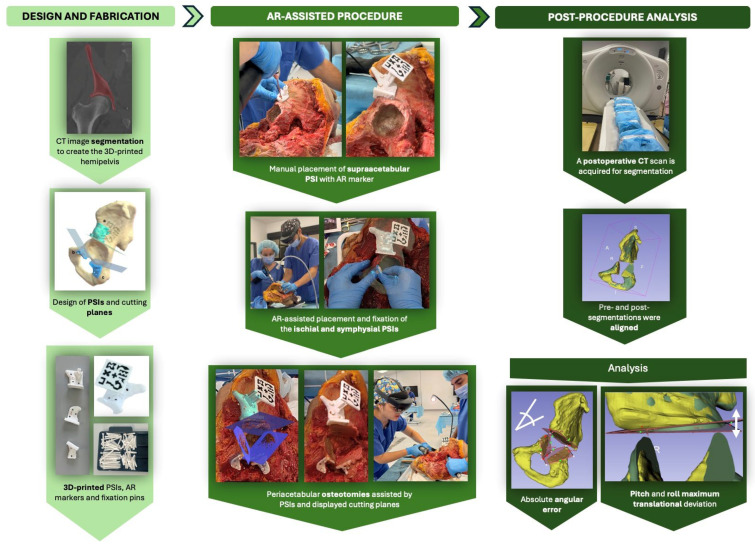
Overview of the AR-assisted osteotomy workflow. The experimental process consisted of three phases: (1) design and 3D printing of patient-specific instruments (PSIs) and AR markers from segmented CT data; (2) AR-assisted surgical procedures on cadaveric hemipelves, including PSI placement and guided osteotomies using a head-mounted display (HoloLens 2); and (3) post-procedural CT-based analysis to assess angular and translational accuracy by aligning pre- and post-operative segmentations.

The experimental workflow began with the selection of ten left-sided cadaveric hemipelvis specimens. The workflow was organized into three main phases: (1) the design and fabrication of PSIs and AR markers, (2) the AR-assisted surgical experiment, and (3) post-procedural CT-based evaluation (Figure 2).

For the first phase, pre-operative CT scans were acquired for all fresh-frozen specimens. The images were then segmented to allow for the design and 3D printing of patient-specific instruments (PSIs) for guiding peri-acetabular osteotomies by the bioengineering team.

During phase 2, the experiment was carried out by two surgical teams, designated as Team A and Team B. Each team was composed of two surgeons: one experienced surgeon who performed the procedure and one assistant who supported intraoperative tasks. Team A was responsible for the odd-numbered cadavers, while Team B performed the even-numbered cases. In addition to the surgical staff, each team included two engineers: a software engineer and a biomedical engineer. The software engineer supervised the experiment through the display of the HMD on a second laptop screen and provided technical assistance with the AR application and the HoloLens 2 display, while the biomedical engineer managed data collection and experimental evaluation throughout the procedure.

**Figure 3:**
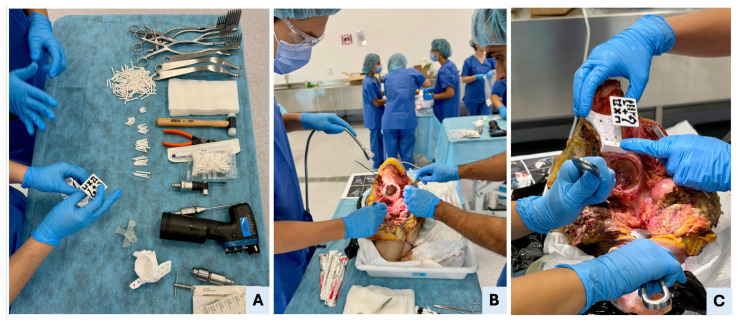
Surgical setup, hemipelvectomy approach, and manual PSI placement. (**A**) Overview of the surgical table with all necessary tools, including standard cutting instruments (oscillating saw, surgical blades, chisels, and drill), soft tissue retractors, fixation pins, 3D-printed patient-specific instruments (PSIs), and AR marker. The surgeon is verifying the AR marker’s correct fit within the supraacetabular PSI’s dedicated socket. (**B**) Execution of a standard oncological T-shaped hemipelvectomy approach in Cadaver II to expose the pelvic anatomy. (**C**) Manual placement of the supraacetabular PSI demonstrates precise fit to the bony relief; accurate positioning of the AR marker ensures reliable registration for holographic overlay in the mixed-reality environment.

**Figure 4:**
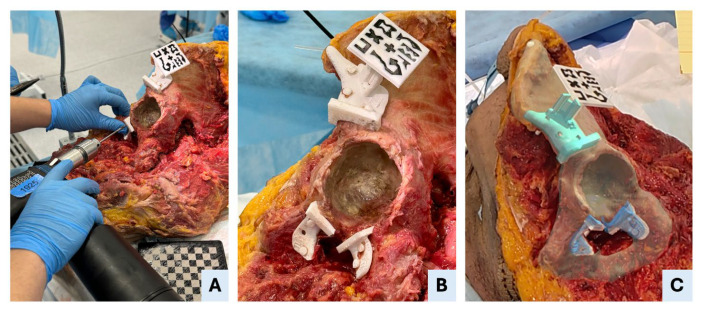
AR-assisted positioning and fixation of patient-specific instruments (PSIs). (**A**) Drilling through the fixation holes of the symphysial PSI to secure the guide to the bone. (**B**) An intraoperative view shows that all three PSIs—supraacetabular, ischial and symphysial—are accurately positioned and fixed in specimen II. (**C**) Mixed-reality view as seen through the head-mounted display, illustrating the superimposition of the holographic PSIs onto the real anatomical structures, enabling precise intraoperative guidance.

**Figure 5:**
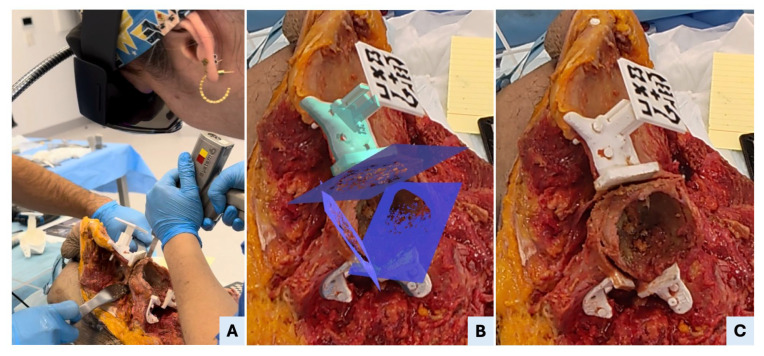
AR-assisted osteotomy execution. (**A**) Surgeon performing the supraacetabular osteotomy using an oscillating saw, guided by both the physical support of the PSI and the visual alignment provided by the AR-displayed cutting plane. (**B**) A real-time screenshot from the head-mounted display (HMD) shows the mixed-reality interface with the three planned osteotomy planes superimposed on the surgical field, as visualized by the surgeon during the procedure. (**C**) Post-osteotomy view showing the supraacetabular PSI remaining securely fixated to the bone, confirming the guide’s stability throughout sawing. The osteotomy surfaces appear clean and well-aligned, reflecting the precision enabled by the combined physical and AR guidance.

Each procedure began with a standard oncological T-incision for an internal hemipelvectomy approach (Figure 3). Following this, the supraacetabular PSI was manually positioned and fixed, and the AR marker was attached to it (Figure 3). The remaining PSIs were then aligned and fixed using the holographic guidance displayed through the HoloLens 2 (Figure 4). Once all guides were secured, the osteotomies were performed using both the physical PSIs and the superimposed cutting planes for assistance (Figure 5). After completing the resections, the cadavers’ soft tissues were sutured, and the specimens were refrozen. Once frozen, they were mobilized again to perform the postoperative CT scan required for evaluation.

### 2.3. Analysis

**Figure 6:**
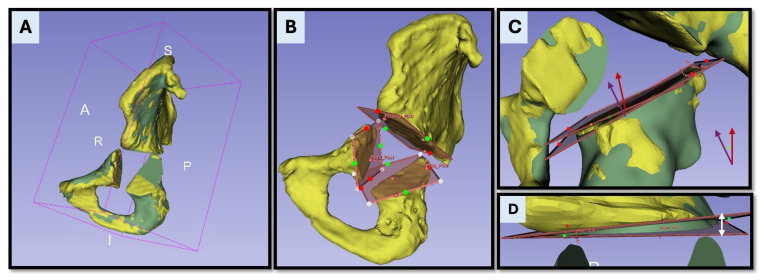
Postoperative accuracy analysis based on CT registration and osteotomy comparison. (**A**) Postoperative (yellow) and preoperative (green) CT segmentations are rigidly aligned within a unified coordinate system to enable spatially accurate comparison. The postoperative model is transformed into the planning coordinate system, which serves as the ground truth for analysis. (**B**) Visualization of the osteotomy planes as reconstructed from the postoperative CT data. (**C**) Superimposition of the planned and executed osteotomy planes, with their respective normal vectors (planned—purple and executed—red). The absolute angular deviation is calculated as the angle between these vectors. (**D**) Maximum translational deviation is determined as the largest linear distance between the planned and executed planes, measured at the point of greatest discrepancy along the osteotomy surface. This distance is visually indicated by the white arrow.

After the experiment, both bone and surgical elements were segmented from the postoperative CT scans. These segmentations were then aligned with the preoperative CT data by registering both datasets within a common coordinate system (Figure 6A). This was achieved through a combination of manual alignment and automated registration using the Iterative Closest Point (ICP) algorithm, resulting in a final transformation matrix. This process ensured consistent spatial referencing between planned and executed surgical data.

The post-procedural evaluation included three main components: osteotomy precision, task time, and user perception.

**Figure 7:**
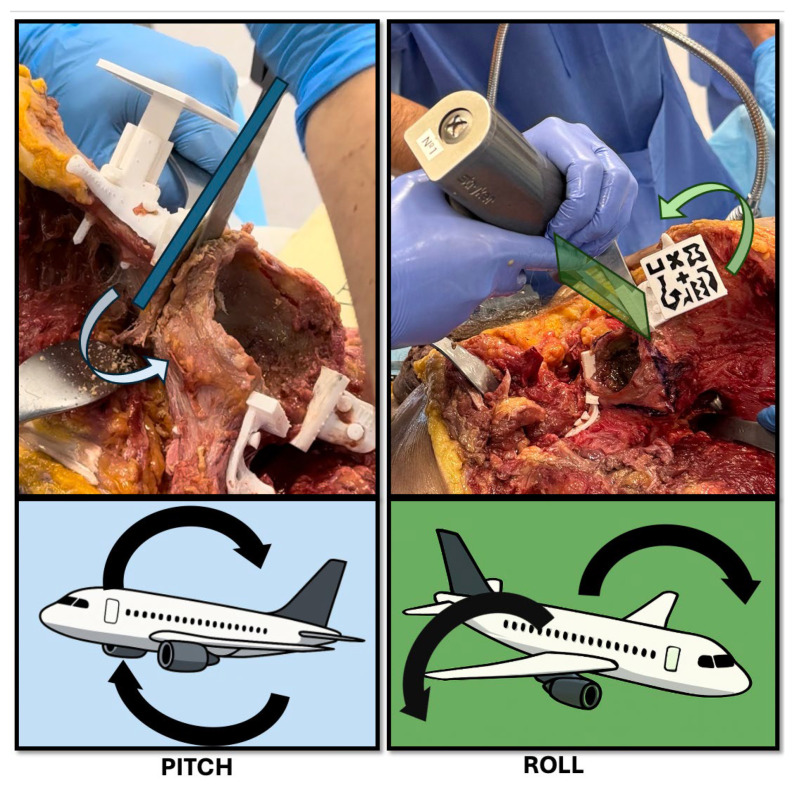
Schematic representation of pitch and roll deviations during osteotomy execution. The top panels show intraoperative examples of potential angular deviations using a surgical saw. (**Left**) (Pitch): forward or backward tilting of the saw in the sagittal plane, resulting in a change in osteotomy angle relative to the planned trajectory. (**Right**) (Roll): medial or lateral tilting of the saw in the coronal plane, causing off-axis resection and possible asymmetry. Bottom panels use aircraft analogies to illustrate the same concepts: pitch corresponds to nose-up or nose-down movements, and roll reflects side-to-side wing rotation. These angular deviations are critical parameters in evaluating the precision of AR-assisted bone cuts.

Osteotomy accuracy was evaluated through two main measures: the absolute angular error and the maximum translational deviation. First, the absolute angular error was calculated by extracting the normal vectors of the planned and executed osteotomy planes and measuring the angle between them (Figure 6C). This angular difference was initially computed in radians and converted to degrees (°) to enhance clinical interpretability. This value reflected the angular deviation introduced during osteotomy execution in absolute terms. Second, the maximum translational deviation (MTD) was calculated as the largest linear distance between the planned and executed osteotomy planes (Figure 6D). This measurement was performed after aligning postoperative CT segmentations with preoperative models using a rigid registration method based on the Iterative Closest Point (ICP) algorithm. The deviation was assessed at the osteotomy corner point, which represents the most clinically relevant location in oncologic resections. MTD was computed separately in the sagittal plane (pitch) and the coronal plane (roll) to capture directional deviations of the cutting trajectory (Figure 7). Task time was recorded for each procedural step, including the PSI placement and fixation, as well as osteotomy execution.

Accuracy thresholds were defined as follows: for absolute angular error, optimal (0–5°), acceptable (5–10°), and non-acceptable (>10°); for maximum translational deviation, excellent (0–3 mm), optimal (4–6 mm), acceptable (7–10 mm), and non-acceptable (>10 mm).

To further assess osteotomy accuracy, two comparative analyses were conducted: (A) between the executed osteotomy planes and the initial preoperative plans, and (B) between the executed planes and the AR-displayed cutting planes following supraacetabular PSI placement. Comparison A reflects the overall deviation from the original plan, encompassing cumulative errors from PSI positioning, AR guidance, and osteotomy execution itself. In contrast, Comparison B isolates the error associated primarily with PSI placement, as it evaluates deviations from the AR-displayed planes immediately after guide positioning. This two-tiered approach enables the identification of potential intermediate errors introduced during the workflow. This distinction helps clarify the relative contribution of assisted PSI-positioning to the final surgical accuracy. A Wilcoxon signed-rank test was used to detect significant differences between the two comparisons, and a Bland-Altman plot analysis was generated to assess agreement between the measurements.

User feedback was gathered to assess the usability and practical value of the AR-assisted workflow. Surgeons provided subjective evaluations focusing on the interface’s intuitiveness, clarity of holographic guidance, and its influence on efficiency and confidence during osteotomy execution.

These analyses allowed evaluation of the combined effectiveness of the dual PSI placement guidance and real-time holographic cutting plane display, validating the feasibility and precision of the AR-guided workflow in a clinical scenario.

## 3. Results

### 3.1. Osteotomy Accuracy

#### 3.1.1. Absolute Angular Error

Table 1 reports the absolute angular error (°) for each osteotomy site across the ten cadaveric specimens. The mean angular error with 95% confidence intervals was 4.58 ± 2.12° for the supraacetabular osteotomy, 5.69 ± 3.71° for the ischial osteotomy, and 5.08 ± 2.35° for the symphysial osteotomy. Overall, 86.66% of osteotomies were performed within the predefined acceptable angular threshold, with a pooled 95% confidence interval of 5.11 ± 1.43° (Table 2).

#### 3.1.2. Maximum Translational Deviation

The maximum translational deviation (MTD) was assessed for each osteotomy in both the sagittal (pitch) and coronal (roll) planes. Pitch deviation (pMTD) refers to errors in the cutting direction—whether the saw is tilted upward or downward—while roll deviation (rMTD) reflects medial-lateral inclination, i.e., tilting the saw left or right. MTD values for pitch and roll are presented in Table 3 and Table 4, respectively.

In terms of pitch, the mean MTD with 95% confidence intervals was 5.49 ± 2.37 mm for the supraacetabular osteotomy, 4.95 ± 3.29 mm for the ischial osteotomy, and 3.14 ± 1.53 mm for the symphysial osteotomy. Overall, 93.33% of osteotomies fell within the predefined acceptable translational threshold, with a pooled pMTD of 4.53 ± 1.32 mm (Table 5).

Roll deviations showed improved accuracy, with a mean MTD of 3.12 ± 1.18 mm for the supraacetabular osteotomy, 2.88 ± 1.75 mm for the ischial osteotomy, and 2.38 ± 1.21 mm for the symphysial osteotomy. All osteotomies (100%) remained within the acceptable threshold, with a pooled mean rMTD of 2.79 ± 0.72 mm (Table 6).

### 3.2. Task Times

The following table (Table 7) presents the time required to complete each task, including PSI positioning and fixation, as well as osteotomy execution for each anatomical site. On average, each specimen required 8.57 ± 1.14 min (mean ± 95% CI) to complete all tasks across the three osteotomy sites.

### 3.3. Agreement Analysis

To further evaluate osteotomy accuracy, angular deviation was compared between two stages: (A) the final executed osteotomy planes and the initial preoperative plans, and (B) the executed planes and the AR-displayed cutting planes following PSI placement. The Wilcoxon signed-rank test revealed statistically significant (*p* < 0.05) differences between comparisons A and B for all anatomical sites, with *p*-values of 0.013 for the supraacetabular osteotomy, 0.002 for the ischial, and 0.002 for the symphysial osteotomy.

**Figure 8:**
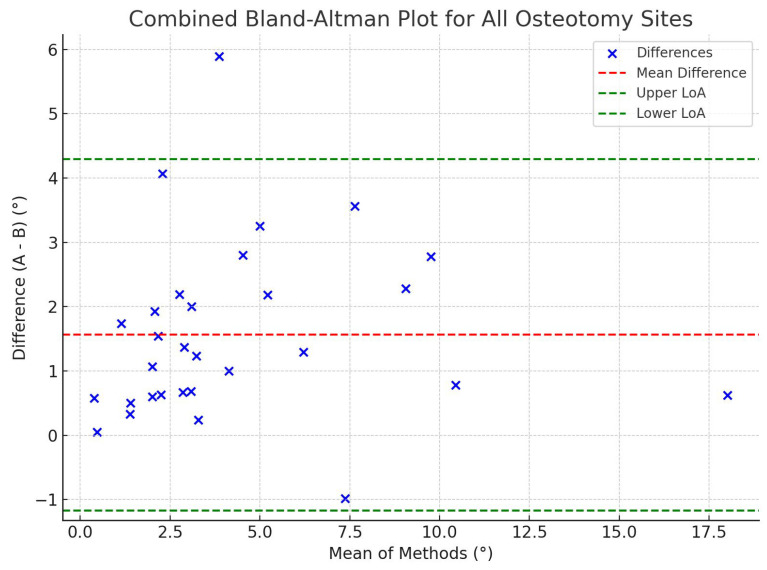
Combined Bland–Altman plot comparing angular deviations from the planned osteotomy plane (A) and the AR-displayed plane after PSI placement (B). The plot shows the mean of the two measurements on the *x*-axis and their difference (A–B) on the *y*-axis, combining all anatomical sites (supraacetabular, ischial, and symphysial). The red dashed line represents the mean difference, while the green dashed lines indicate the limits of agreement (±1.96 SD). Most data points fall within the boundaries of agreement, suggesting overall consistency between the two measurements, though Wilcoxon analysis confirmed a statistically significant difference favoring greater precision in B (*p* < 0.05).

These results indicate that angular deviations were significantly smaller in Comparison B, demonstrating that AR guidance contributed to highly accurate PSI positioning. The combined Bland–Altman plot (Figure 8) supports this observation, showing a consistent positive bias: deviations from the initial plan (A) were greater than deviations from the AR-displayed planes (B).

Additionally, comparison between odd- and even-numbered specimens showed no statistically significant differences for any anatomical site (supraacetabular: *p* = 0.81; ischial: *p* = 0.19; symphysial: *p* = 0.06), indicating that both surgeons performed consistently and introduced no systematic bias into the workflow.

### 3.4. User Perception

Users reported high satisfaction with the implemented AR-assisted system in 80% of the cases. (Table 8). Surgeons emphasized the ease of use, intuitive learning curve, ergonomic integration, and positively evaluated the software interface design and image quality. The visualization of cutting planes was particularly noted as a valuable clinical feature. However, some limitations were identified, including occasional inaccuracies in depth perception, which appeared to vary depending on the angle of vision. In addition, button precision was suboptimal and highlighted as an area for future improvement.

## 4. Discussion

### 4.1. Phased Challenges, Adverse Outcomes, and Outliers

During the study, several adverse events and technical challenges were encountered, some of which likely contributed to the observed outliers in angular or translational accuracy. These issues were categorized as material-related, software-related, hardware-related, or anatomical, and are detailed below.

Material-related complications included hardware fragility and instability. In specimen III, the supraacetabular socket experienced structural breakage; a temporary repair was implemented, and the osteotomies were executed successfully with satisfactory accuracy. In specimen V, the same socket appeared macroscopically unstable due to a loose fit, yet it functioned adequately during the procedure. In specimen IX, a marker cube fractured, but a backup was available, allowing the surgery to proceed without loss of guidance accuracy, although other factors contributed to suboptimal results in the supraacetabular osteotomy in that case. Additionally, a minor detachment of the marker cube from its base occurred, but it was quickly resolved using a replacement component that we had as a backup.

A segmentation error was noted in specimen IV, where the 3D reconstruction around the acetabulum did not fully correspond to the anatomical reality. Despite this discrepancy, the printed guides were used as planned. Thanks to the proper placement of the PSIs, the osteotomies were executed successfully, and acceptable accuracy was achieved, underscoring the robustness of the PSI-based workflow even when minor preoperative modelling errors exist.

A mechanical complication occurred in specimen IX, where a fixation pin fractured. The pin was replaced promptly without further incidence or impact on osteotomy accuracy.

Regarding instrumentation, the study employed four oscillating saw motors: two high-performance surgical-grade devices and two osteosynthesis motors with lower torque and thinner blades. Due to battery depletion during prolonged operating times, specimens VI through IX were operated using the lower-powered motors. These devices produced greater saw oscillation and insufficient cutting stability, particularly in osteotomies involving large bony contact surfaces, which likely accounts for several outliers in those cases.

Head-mounted display (HMD) overheating was encountered during prolonged use, particularly in specimens V and VII, after approximately three hours of continuous operation (from specimen I to V). Procedures had to be paused temporarily for 10 min to allow cooling. The experiment was conducted during the summer, and high ambient temperatures may have further contributed to the issue.

Software-related incidents were rare but notable. In specimen VI, the application initially loaded an incorrect cadaver dataset, which was resolved by restarting the system. A similar error occurred in specimen IX, where the AR overlay was inverted (the inferior part was shown superior and vice versa). Again, this was corrected through an application reset without affecting the surgical workflow.

Finally, cadaver-related anomalies were noted in multiple cases. In specimen X, an undisplaced acetabular fracture, not identified during CT planning, was discovered intraoperatively. However, this did not impair the accuracy of PSI placement or osteotomy execution. Additionally, bone quality appeared unusually poor in specimens VII and VIII, likely due to partial unfreezing and underlying sclerosis. This condition may have contributed to reduced cutting precision and the outliers observed in those two cases.

Despite these challenges, most procedures proceeded with minimal impact on surgical precision, and the system demonstrated resilience in the face of both technical and anatomical variability.

### 4.2. Strengths and Limitations

A key strength of this study lies in its high clinical realism. All procedures were performed on cadaveric specimens with preserved soft tissues, replicating the mechanical complexity and anatomical variability of real surgical settings. This model enabled the assessment of AR-assisted workflows under conditions that closely mirror the intraoperative environment.

The experimental design replicated realistic intraoperative challenges—such as marker detachment, socket instability, and hardware failure—further validating the system’s robustness. Fixation pins performed reliably, with only one breakage. Notably, there was no statistically significant difference between odd- and even-numbered specimens, confirming consistent performance between surgeons and absence of operator bias. The inclusion of backup markers and PSIs proved essential and is recommended for clinical implementation.

The primary limitations of this study include the small number of specimens (n = 10) and the absence of a comparative control group (e.g., freehand or conventional PSI workflows). While these factors limit the generalizability of the findings, the sample size was deemed sufficient to establish the feasibility of the AR-assisted workflow. The observed accuracy results were within predefined thresholds and in line with previous reports.

Other investigations, such as those by Sallent et al. and Mediavilla et al., have shown that PSIs improve osteotomy accuracy over freehand techniques, and AR support further enhances this precision [9,22]. As part of our study group, Iribar-Zabala et al. published a preliminary proof-of-concept assessment for the AR software and workflow used in the present study. Their results demonstrated promising accuracy and positive clinician feedback, reinforcing the precision achievable with AR-guided PSI placement (with an overall mean absolute angular value of 2.20° and a mean distance error of 1.19 ± 0.53 mm) [19]. In contrast, studies like Hoch et al. observed higher error rates in AR-assisted Ganz pelvic osteotomies, with angular deviations up to 6°–7° and mean distance errors around 9 mm [16]. Our cadaveric findings reflect a marked improvement over these values. Specifically, we report a mean absolute angular error of 5.11 ± 1.43°, with 86.66% of osteotomies falling within the predefined acceptable angular threshold. Translational accuracy was also favorable, with a mean pitch maximum translational deviation (pMTD) of 4.53 ± 1.32 mm and a roll MTD (rMTD) of 2.79 ± 0.72 mm. Notably, 93.33% of osteotomies met the predefined pitch accuracy criteria, and 100% met roll criteria, further validating the consistency and reliability of the AR-assisted execution. These findings were supported by the Wilcoxon test, which showed significantly lower angular deviation when comparing the executed planes to the AR-displayed planes after PSI placement (*p* = 0.013, 0.002, and 0.002 for supraacetabular, ischial, and symphysial sites, respectively). This highlights the enhanced precision introduced by the AR in the earlier step of guide placement. Residual error appears to originate mainly from the osteotomy execution phase, influenced not only by AR assistance but also by mechanical factors such as saw stability and surgeon dexterity.

Moreover, AR integration has been explored in other pelvic applications. Ogawa et al. and Tsukada et al. reported angular errors between 2.1° and 2.7° using AR systems for acetabular component placement [23,24], and Kimura et al. confirmed improved targeting within ±5° margins using a pin-less AR approach [17]. Although these studies pertain to arthroplasty, they illustrate the broad utility of AR in pelvic surgery. In the oncologic context, Wang et al. reported mean osteotomy errors of 2.66 mm and 2.16° using fluoroscopy-calibrated PSIs [20], further supporting the accuracy potential of image-guided techniques. Our results are comparable, with the added advantage of real-time visualization through a head-mounted display (HMD), eliminating the need for fluoroscopy and reducing radiation exposure. Notably, our angular deviations are reported as absolute 3D errors, capturing discrepancies across all spatial planes, which explains the slightly higher values in absolute terms when compared to single-axis reports. Additionally, we focused on maximum rather than mean translational deviation, offering a more stringent and clinically relevant measure for oncologic resections, where ensuring clear margins at the osteotomy corners is critical.

Unlike conventional navigation systems, which require optical tracking and unobstructed marker visibility, AR-based solutions—especially those using HMDs—offer more ergonomic, uninterrupted visualization. Navigation systems, though accurate, are often cumbersome and time-consuming to set up [6]. Smartphone- and tablet-based AR are portable but require manual handling, limiting dexterity [12,25]. In contrast, HMDs provide hands-free, real-time guidance from the surgeon’s perspective, enhancing workflow integration and surgical precision [13]. In our case, the AR software was developed in-house and integrated into a head-mounted display, with the hospital serving as a single point of production for segmentation, guide design, 3D printing, and AR setup—streamlining the workflow and maximizing system autonomy [7,26].

In terms of workflow efficiency, the AR-assisted procedure demonstrated clinically acceptable task times. The mean total time—encompassing both PSI placement and osteotomy execution—was approximately 8.5 min per specimen, with low inter-specimen variability. Importantly, this duration includes the actual cutting time, which is an inherent and unavoidable part of the surgical procedure. Therefore, only a small portion of this time reflects the AR-specific setup or interaction. In contrast, conventional navigation systems often introduce additional setup time—typically between 10 to 30 min—that is not part of the essential surgical task and may delay the overall procedure [6,27]. In our workflow, AR guidance was seamlessly integrated and did not result in meaningful time overhead or disruption. This supports its practicality for intraoperative use without compromising surgical efficiency.

### 4.3. Technology Adoption and Usability

User satisfaction with the AR-assisted workflow was high, with 80% of surgeons rating the system 4 or 5 in overall satisfaction, PSI positioning, and osteotomy execution. Key advantages noted were system intuitiveness, ergonomic integration, and especially the visualization of cutting planes.

Consistent with Verhellen et al., surgeons valued usability and performance but reported minor drawbacks, including minor visual discomfort after extended use, occasional depth perception issues, and suboptimal button precision—common limitations associated with HMD-based systems [27,28].

Despite these, users found the system clinically valuable and expressed strong interest in future adoption, contingent on minor hardware and interface improvements.

## 5. Conclusions

This cadaveric study confirms the feasibility, accuracy, and clinical usability of an augmented reality (AR)-assisted workflow for patient-specific instrument (PSI) positioning and osteotomy execution in complex pelvic tumor resections. The in-house–developed AR system, delivered through a head-mounted display (HMD) and fully integrated within a hospital-based production pipeline, provided dual-function intraoperative guidance with encouraging results.

Despite encountering technical and anatomical challenges, 86.66% of osteotomies achieved angular accuracy within the predefined acceptable threshold. Translational deviations also remained within clinically relevant limits in 93.33% of cases for pitch and 100% for roll orientations, based on stringent maximum deviation criteria. The AR system contributed substantially to precise PSI positioning, while most residual deviations were attributed to execution-phase factors such as saw control and minor depth misalignment.

Surgeons rated the system as intuitive and clinically beneficial, reinforcing its potential for future adoption in oncologic workflows. With continued refinements in ergonomic design and interface responsiveness, AR-guided surgery offers a reliable, accurate, and user-friendly solution for enhancing precision in complex orthopedic interventions.

## Figures and Tables

**Table 1:** Absolute angular deviation between planned and executed osteotomy planes for each anatomical site.

Specimen	Supraacetabular (°)	Ischial (°)	Symphysial (°)	Supraacetabular (Radians)	Ischial (Radians)	Symphysial (Radians)
I	3.85	2.01	5.92	0.07	0.04	0.10
II	3.41	6.31	6.86	0.06	0.11	0.12
III	2.56	3.84	3.58	0.04	0.07	0.06
IV	4.64	1.65	9.42	0.08	0.03	0.16
V	2.94	3.19	0.49	0.05	0.06	0.01
VI	6.88	10.84	2.54	0.12	0.19	0.04
VII	0.68	2.30	3.42	0.01	0.04	0.06
VIII	6.62	18.32	10.20	0.12	0.32	0.18
IX	11.15	4.10	1.55	0.19	0.07	0.03
X	3.03	4.32	6.81	0.05	0.08	0.12

Angular error was calculated in radians between the normal vectors of the planned and executed planes and converted to degrees (°) for clinical interpretation. Both units are shown per specimen and anatomical site (supraacetabular, ischial, and symphysial).

**Table 2:** Distribution of absolute angular error across osteotomy sites categorized by accuracy thresholds (in degrees).

Absolute Angular Error	Supraacetabular	Ischial	Symphysial	Total	%
Optimal (0–5°)	7	7	5	19	63.33
Acceptable (5–10°)	2	1	4	7	23.33
Non-acceptable (>10°)	1	2	1	4	13.33

Values represent the number of osteotomies within each accuracy category per anatomical site. Percentages are calculated relative to the total number of osteotomies (n = 30).

**Table 3:** Pitch maximum translational deviation (pMTD) and osteotomy length (pOL) for each specimen and anatomical site.

Specimen	Supraacetabular		Ischial		Symphysial	
	pMTD	pOL	pMTD	pOL	pMTD	pOL
I	4.72	70.20	1.91	54.29	4.16	40.16
II	4.22	70.83	5.26	47.54	4.34	36.45
III	3.10	69.27	3.77	56.16	2.66	42.44
IV	5.02	61.89	1.61	55.95	5.82	35.06
V	3.62	70.50	3.37	60.38	0.35	40.99
VI	8.14	67.50	8.43	44.02	1.58	35.62
VII	0.90	75.42	1.68	41.80	1.28	21.48
VIII	8.83	76.09	16.71	50.48	6.75	37.50
IX	12.29	62.37	3.28	45.72	1.04	38.39
X	4.05	76.56	3.45	45.72	3.43	28.74

pMTD values (in mm) represent the maximum translational error at the osteotomy corner point in the sagittal plane (pitch), indicating upward or downward deviation of the saw. pOL refers to the measured length of each osteotomy in the analyzed plane (sagittal) and is provided to contextualize the deviation.

**Table 4:** Roll maximum translational deviation (rMTD) and osteotomy length (rOL) for each specimen and anatomical site.

Specimen	Supraacetabular		Ischial		Symphysial	
	rMTD	rOL	rMTD	rOL	rMTD	rOL
I	3.16	46.92	1.25	35.53	3.43	33.12
II	2.72	45.61	3.44	31.12	2.55	21.16
III	1.82	40.70	2.07	30.81	1.94	30.98
IV	3.39	41.78	0.90	31.40	4.59	27.67
V	2.35	45.82	1.98	35.54	0.27	32.00
VI	4.41	36.59	5.03	26.26	1.37	30.79
VII	0.35	29.54	0.95	23.59	1.00	16.70
VIII	4.86	41.90	8.87	26.78	5.46	30.35
IX	6.03	30.59	1.77	24.70	0.74	27.32
X	2.12	40.12	2.54	33.57	2.44	20.47

rMTD values (in mm) represent the maximum translational error at the osteotomy corner point in the coronal plane (roll), indicating medial or lateral inclination of the saw. rOL refers to the measured length of each osteotomy in the analyzed plane (coronal), which corresponds to the shortest axis and is provided to contextualize the deviation.

**Table 5:** Distribution of pitch maximum translational deviation (pMTD) across osteotomy sites categorized by accuracy thresholds (in mm).

pMTD	Supraacetabular	Ischial	Symphysial	Total	%
Excellent (0–3 mm)	3	7	6	16	53.33
Optimal (4–6 mm)	4	1	4	9	30
Acceptable (7–10 mm)	2	1	0	3	10
Non-acceptable (>10 mm)	1	1	0	2	6.67

Values represent the number of osteotomies falling within each accuracy category per anatomical site. Percentages are calculated relative to the total number of osteotomies (n = 30).

**Table 6:** Distribution of roll maximum translational deviation (rMTD) across osteotomy sites categorized by accuracy thresholds (in mm).

rMTD	Supraacetabular	Ischial	Symphysial	Total	%
Excellent (0–3 mm)	7	8	8	12	76.67
Optimal (4–6 mm)	3	1	2	6	20
Acceptable (7–10 mm)	0	1	0	1	3.33
Non-acceptable (>10 mm)	0	0	0	0	0

Values represent the number of osteotomies falling within each accuracy category per anatomical site. Percentages are calculated relative to the total number of osteotomies (n = 30).

**Table 7:** PSI positioning and osteotomy execution times (in seconds) for each specimen and anatomical site.

Specimen	Supraacetabular		Ischial		Symphysial	
	PSI	Osteotomy	PSI	Osteotomy	PSI	Osteotomy
I	62	80	57	66	77	90
II	78	61	71	65	162	126
III	42	114	68	70	124	63
IV	51	155	88	61	205	23
V	89	55	248	26	115	39
VI	22	224	112	74	66	66
VII	60	30	72	21	22	174
VIII	50	130	231	35	189	55
IX	135	53	85	50	59	35
X	152	36	94	30	126	24
95% CI	74.10 ± 29.42	93.80 ± 44.14	112.60 ± 49.17	49.80 ± 14.35	114.50 ± 42.40	69.50 ± 34.68

Values represent the time (in seconds) required for PSI placement and fixation (PSI) and subsequent osteotomy execution (Osteotomy) for the supraacetabular, ischial, and symphysial regions in each specimen. The final row presents the mean time for each task with its corresponding 95% confidence interval.

**Table 8:** Surgeons’ satisfaction ratings for AR-assisted PSI positioning, osteotomy execution, and overall experience.

Specimen	AR-Assisted PSI Positioning	AR-Assisted Osteotomy Execution	Overall Satisfaction
I	4	4	4
II	3	4	4
III	3	5	4
IV	5	4	5
V	3	3	5
VI	3	4	4
VII	4	5	5
VIII	3	3	3
IX	3	4	3
X	3	5	4
Mean	3.4	4.1	4.1

Scores reflect surgeon-reported satisfaction for each specimen using a five-point Likert scale (1 = very poor, 5 = excellent). The final row shows the mean rating for each category across all specimens.

## Data Availability

Data will be provided upon request from the corresponding author.

## Abbreviations

The following abbreviations are used in this manuscript:

AR	Augmented Reality
ASA	Acrylonitrile Styrene Acrylate
CI	Confidence Interval
CT	Computer Tomography
HMD	Head-Mounted Display
ICP	Iterative Closest Point
MRTK	Mixed-reality Toolkit
MTD	Maximum Translational Deviation
OL	Osteotomy Length
PLA	Patient-Specific Instrument
pMTD	Pitch Maximum Translational Deviation
pOL	Pitch Osteotomy Length
rMTD	Roll Maximum Translational Deviation
rOL	Roll Osteotomy Length
